# Development of a behavioural intervention for cognitive fatigability in multiple sclerosis: Protocol for a pilot and feasibility study

**DOI:** 10.3389/fresc.2022.999266

**Published:** 2022-11-16

**Authors:** Lisa A. S. Walker, Jason A. Berard, Tamanna Islam, Lara A. Pilutti, Sarah A. Morrow, Marcia Finlayson

**Affiliations:** ^1^ Department of Neuroscience, Ottawa Hospital Research Institute, Ottawa, ON, Canada; ^2^ University of Ottawa Brain and Mind Research Institute, University of Ottawa, Ottawa, ON, Canada; ^3^ Department of Psychology, Carleton University, Ottawa, ON, Canada; ^4^ Department of Psychology, University of Ottawa, Ottawa, ON, Canada; ^5^ Interdisciplinary School of Health Sciences, University of Ottawa, Ottawa, ON, Canada; ^6^ MS Clinic, London Health Sciences Centre, London, ON, Canada; ^7^ School of Rehabilitation Therapy, Queen’s University, Kingston, ON, Canada

**Keywords:** multiple sclerosis, cognitive fatigability, fatigue, cognition, neuropsychology, exercise, depression, intervention

## Abstract

**Background:**

Up to 90% of people with multiple sclerosis (PwMS) subjectively report fatigue as one of their worst symptoms. Fatigability is an objectively measured component of fatigue. Cognitive fatigability (CF) is a breakdown in task performance following sustained cognitive effort. There is a paucity of interventions targeting CF in MS. The prior success of behavioural interventions at improving subjective fatigue suggests that their adaptation may yield similar results for CF. Given the relationship between CF, sleep quality, and mood, a behavioural intervention targeting these factors, such as cognitive behavioural therapy (CBT), is warranted. Given the multidimensional nature of fatigue, a multifaceted approach targeting lifestyle factors and coping (e.g., fatigue management education supplemented by CBT for insomnia and exercise) might prove efficacious.

**Aim:**

We describe a protocol for a pilot feasibility study to design and implement a multi-dimensional behavioural intervention to improve CF in PwMS.

**Methods:**

*Stage 1*: development of a multi-dimensional group-based videoconference-delivered behavioural intervention based on a previously successful fatigue management program for PwMS. A facilitator manual will be drafted. Course material will focus on four themes: body (sleep and physical activity), mood (impact of depression and anxiety), mind (cognitive contributions), and context (pacing and communication). *Stage 2*: a needs assessment survey will be completed by 100 PwMS for input on what factors are important contributors to their CF. Modifications will be made to the course material and manual. *Stage 3*: the facilitator-delivered intervention will include 20 PwMS. After baseline assessment, participants will attend weekly 70-min videoconference group sessions for 8 weeks, including homework assignments. Follow-up assessment will re-evaluate outcomes. *Stage 4*: analysis and dissemination of results. The primary outcome is improvement in CF. Additional feasibility outcomes will determine if a randomized control trial (RCT) is pursued. *Stage 5*: refine the intervention based on outcomes and feedback from participants. Determining which aspects participants felt were most effective will help inform RCT design.

**Conclusion:**

The long-term goal is to ensure that PwMS have access to effective interventions in real-world settings to improve quality of life and enhance their ability to participate in cognitively demanding activities that they enjoy.

## Introduction

Fatigue is one of the most commonly reported symptoms in MS, occurring in up to 90% of affected individuals (1, 2), with negative impacts on quality-of-life (3), self-esteem (4), and employability (5, 6). What is meant by the term fatigue varies throughout the literature. Definitions include a state of reduced capacity for work following a period of mental or physical activity (7), a feeling of physical tiredness and lack of energy that is distinct from sadness or weakness (8), extreme tiredness with the feeling that one needs to rest (9), a feeling of lack of energy, weariness, and aversion to effort (10), among other definitions (11). Fatigue has largely been regarded as a subjective experience unique to the individual, and thus it is typically measured by self-report. A number of questionnaires have been developed to that end, with some attempting to quantify fatigue and others evaluating the impact of fatigue on daily functioning, or both (12–17).

**Figure 1:**
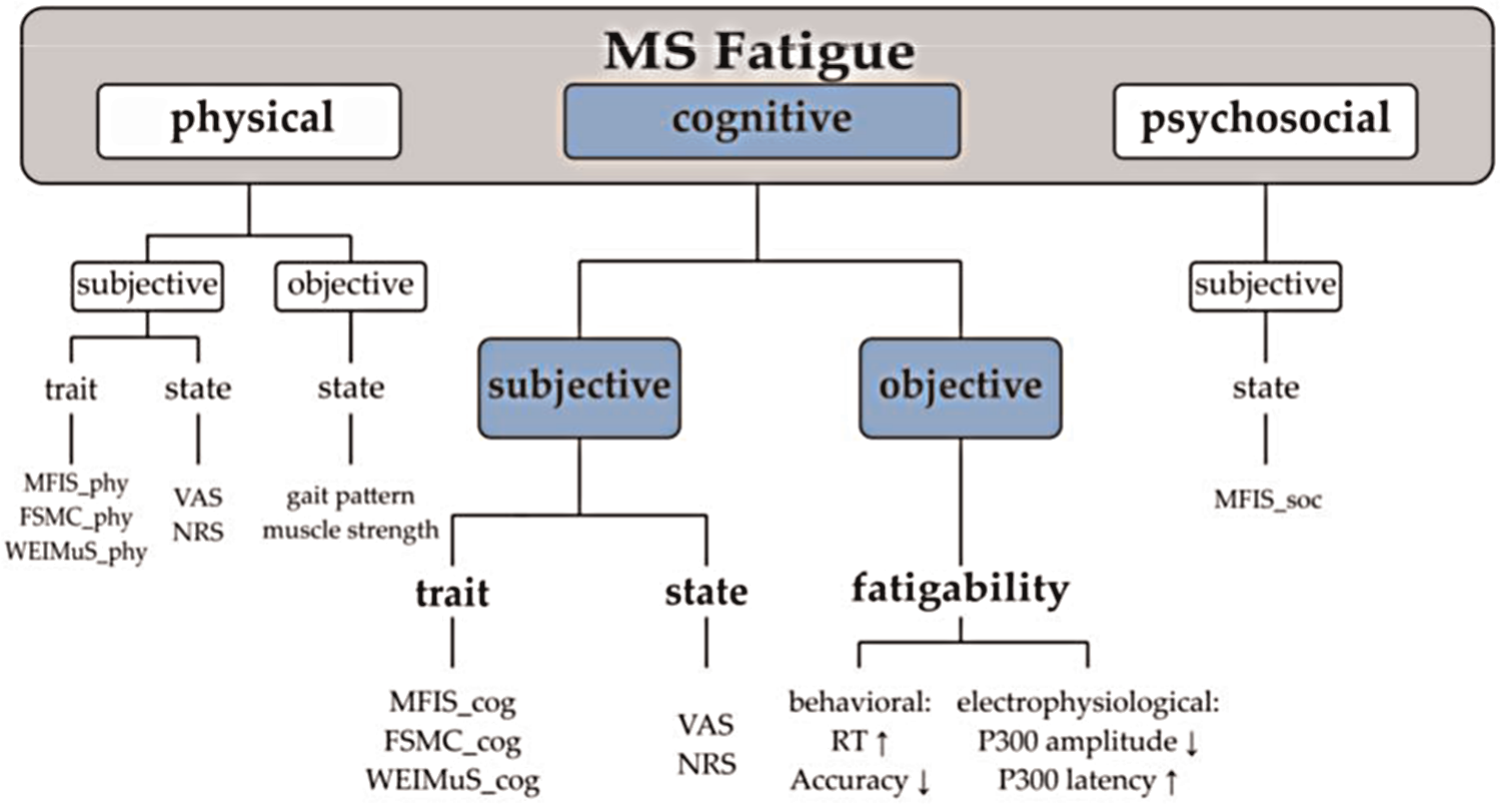
Unified fatigue taxonomy. Fatigue classification. MRS, Modified Fatigue Impact Scale; MS, multiple sclerosis; FSMC, Fatigue Scale for Motoric and Cognitive Functions; WEIMuS, WuerzburiFatigue Inventory for Multiple Sclerosis; VAS, Visual Analogue Scale; NRS, Numerical Rating Scale; RT, reaction time (20).

Given the disparate manner in which fatigue has been addressed in the literature, a unified taxonomy has been posited that distinguishes **fatigue** (i.e., an individual's subjective sensations) from **fatigability** (i.e., objective changes in performance) in order to provide clarification and consistency in both clinical and research applications (18, 19) (Figure 1). While there is a body of research that focuses on physical or motor fatigability (21), the focus of the current project is cognitive fatigability (CF) which can be operationally defined as *an inability to maintain optimal task performance throughout the duration of a sustained cognitive task* (7, 22, 23). Although fatigue is a well-studied concept in MS, CF is less well understood. While most studies measure fatigue using subjective self-report tools, fewer have measured objective CF. Given the negative impact of CF (24, 25), interventions are beginning to be introduced to address this concern, but the field is in its infancy.

Despite the nascent nature of the field, CF has been consistently demonstrated in MS through evidence of a breakdown in task performance across a variety of different cognitive tasks. Those with MS become more cognitively fatigued than healthy controls when performing information processing speed tasks, as reflected by a breakdown in their task accuracy (22, 23, 26). Similarly, reaction time increases over time more so for those with MS compared to controls on simple sustained attention tasks (27–29). Cognitive control (i.e., the combination of processes that allow adaptation of information processing depending on task goals) also declines over time in those with MS (30). Estimates of the frequency of CF is similar to the frequency of cognitive impairment in general in those with MS (31), with *1 in 2* meeting defined criteria for CF in two studies (32, 33).

Our group has extensively evaluated CF in MS. The current project is a natural extension of our work, which initially stemmed from listening to what was important to our patients from our clinical work. They told us that their ability to remain employed was negatively impacted by their susceptibility to fatigue. They noted that their cognitive performance waned as their day progressed but that their employers had little appreciation of this. Thus, it became our goal to find methods of objectively quantifying their experience of declining performance with sustained cognitive effort given that the self-report measures typically used did not capture their experience. Indeed, objectively measured CF does not typically correlate with subjective self-report measures of fatigue (23).

While CF can be evaluated in a number of different ways (34), our group has used the Paced Auditory Serial Addition Test (PASAT) as a measurement tool. Its sensitivity to CF differs depending on how it is scored (23, 26). When CF is evaluated longitudinally, it was found that the magnitude of CF does not change over time early in the disease course (35). Work in the field of CF has also recently been translated into clinical applications with the development of normative data to allow clinicians to determine whether the degree of CF experienced by their patients is statistically significant (36, 37).

Mechanisms of CF have been proposed, although studies vary regarding how CF is measured. CF has been associated with motor and processing speed, gender, and intelligence (33). Sleep quality, and to a lesser extent depression, have also been found to be predictors of CF in MS (38), although others found that the relationship between CF and sleep quality diminishes after controlling for depression (39). It has been postulated that impaired slow wave sleep may be causing the reduced sleep quality that contributes to CF and so treatments improving non-REM efficiency may be warranted (33). CF may also reflect brain-derived fatigue (i.e., directly related to pathological processes in the brain). In MS, this has been termed primary fatigue (40, 41). Disruptions in circuits involving the basal ganglia, frontal cortex, and thalamus have been implicated (42), and lesions in attention and arousal pathways, reticular and limbic systems and basal ganglia have been associated with CF (43, 44). Biomarkers of CF have also been identified in those with MS *via* functional neuroimaging, with differences in activation patterns in the attention network noted between those with MS and healthy controls before, during, and after a cognitively fatiguing task (29) and connectivity differences between those who subjectively report fatigue and those who do not (40). In addition to the influence of structural disease pathology, there are other biological variables such as inflammation that impact brain-derived central fatigue. Indeed, the influence of pro-inflammatory cytokines on fatigue has been demonstrated in MS (45).

There is an extensive literature addressing the treatment for subjectively evaluated fatigue in MS. These interventions generally stem from three different treatment approaches: pharmacological, procedural, and behavioural. *Pharmacological* treatments are often in the form of stimulants such as methylphenidate. The treatment is presumed to be effective given that it helps to overcome attentional difficulties and slowed processing speed, presumably due to its role as a dopamine agonist (46). Amantadine (antiviral), pemoline (stimulant) and modafinil have demonstrated positive results in treating subjective fatigue in those with MS. *Procedural* interventions include techniques such as light therapy, biofeedback, and neuromodulation. A recent review of the use of transcranial magnetic stimulation in MS found preliminary evidence of a beneficial impact on fatigue but replication in well-designed RCTs was recommended (47).

*Behavioural* approaches to treating fatigue have included a variety of methods including psychotherapy [i.e., cognitive behavioural therapy (CBT) or mindfulness], education/symptom management, cognitive rehabilitation and exercise (48, 49). CBT for insomnia (CBT-I) administered to those with MS has shown efficacy in improving insomnia, subjective fatigue and depression (50). A systematic review and meta-analysis on the utility of mindfulness training in the treatment of fatigue after stroke, TBI and MS concluded that these techniques were moderately effective (51). Another systematic review evaluating the efficacy of patient education programs at improving MS-related fatigue found that these programs had a positive effect, but they stressed the need for multidimensional approaches given that fatigue itself is a multidimensional symptom (52). Exercise training has also been explored as a potentially effective treatment and research in MS suggests that this approach is associated with a moderate reduction in fatigue symptoms (48, 53).

As noted above, while intereventions exist targeting subjective fatigue, our group's systematic review (54) highlighted the paucity of interventions specifically targeting objectively evaluated CF in MS. It is this gap in the literature that led to the current project. The presence of subjectively measured cognitive fatigue has been demonstrated to be predictive of MS disease progression in the form of relapses and brain atrophy (55). As such, early detection and treatment of CF may also have implications for disease course. If we can improve CF, then perhaps we can positively influence both quality-of-life and long-term disease outcomes. While procedural [i.e., transcranial direct current stimulation (tDCS)] (56) and pharmacological (i.e., Fampridine) (57) interventions have been recently studied, to date no behavioural interventions exist to improve CF. Given the negative impact of cognitive impairment (58) and fatigue (59) on quality-of-life, there is a need to find feasible and effective treatments to better the lives of those affected by MS. Although *no studies have yet addressed the impact of behavioural interventions on objective CF specifically*, the positive impact of these interventions on subjective fatigue can provide some direction in potentially fruitful options to pursue. Given the relationship between CF, sleep quality (39) and mood (38) a behavioural intervention designed to target sleep quality and mood, such as CBT, or more specifically CBT-I (50), may be warranted. However, as previously suggested, one must consider the multidimensional nature of fatigue when planning and designing treatment interventions and thus a multi-faceted approach targeting lifestyle factors and coping techniques (e.g., fatigue management education supplemented by elements of CBT-I and exercise) might have a greater chance of efficacy. A meta-analysis of exercise, education, and medication treatment interventions for fatigue noted that exercise interventions appeared to have stronger effects than medication given their ability to assist people with MS in coping with their existing disabilities beyond just symptom control (49), further supporting the inclusion of exercise training as a component of multimodal CF management. As such, this project provides a crucial first step towards establishing a multi-dimensional behavioural intervention as a feasible and effective tool to improve CF in those with MS and provides a foundation upon which to plan a future definitive RCT.

To summarize, in this protocol paper we report on the process of protocol development and the details for our behavioural intervention to target CF in people with MS (PwMS). This rationale stems from the well-developed literature outlining the development and administration of interventions to target fatigue in MS. Behavioural approaches have successfully reduced subjectively reported fatigue (52, 60) where pharmacological treatment of objective CF has shown little effect (57). Studies have demonstrated that CF can be influenced by other variables such as mood and sleep quality (38). There is the potential that additional variables, such as exercise, may also impact objective CF given the promising results observed with subjective fatigue and cognition (61). Through this accumulated evidence, the importance of addressing CF from a varied perspective has been established. The current project is a logical next step as the proposed behavioural intervention is designed to address CF using a multidimensional approach. The aims of the project we are reporting on here in this protocol paper are to develop, and pilot test (test the feasibility and preliminary efficacy of) a behavioural intervention designed to improve CF in persons with MS. In the current paper, we report on the process of protocol development, prior to actual implementation of the intervention. This will involve taking elements of already established fatigue management programs and adapting them to include treatment of factors that are known to contribute to CF (i.e., sleep quality, mood), as well as emerging treatments (i.e., exercise) expected to improve CF based on preliminary research. Clinical experience of the investigators and the perspectives of PwMS will also serve to inform the intervention's design.

## Materials and methods

We will apply for study approval through the Ottawa Health Sciences Network Research Ethics Board.

This is a pilot study that addresses questions of *feasibility* (i.e., whether something can be done, should we proceed with it, and if so, how), as well as a specific design feature where we test the *efficacy* of the intervention on a smaller scale in preparation for a future RCT (62). This project will proceed in five stages.

### Stage 1: Development

Stage 1 concerns the *development* of the multi-dimensional group-based behavioural intervention for CF. Pursuing behavioural treatment options is important given that they have the potential to be easily disseminated, can be made widely accessible, and have demonstrated efficacy at ameliorating secondary symptoms of MS (60). The intervention will be based on a previously successful teleconference-delivered fatigue management program for people with MS that was performed by our group (60). The current method of administration *via* videoconference was chosen given that it is easily accessible, does not require specialized technical support, as well as allowing more face-to-face interaction and therefore has the potential for wide dissemination. A videoconference-delivered program is also timely given that the COVID-19 pandemic has necessitated the need for healthcare professionals to find new and innovative ways of delivering healthcare remotely.

**Figure 2:**
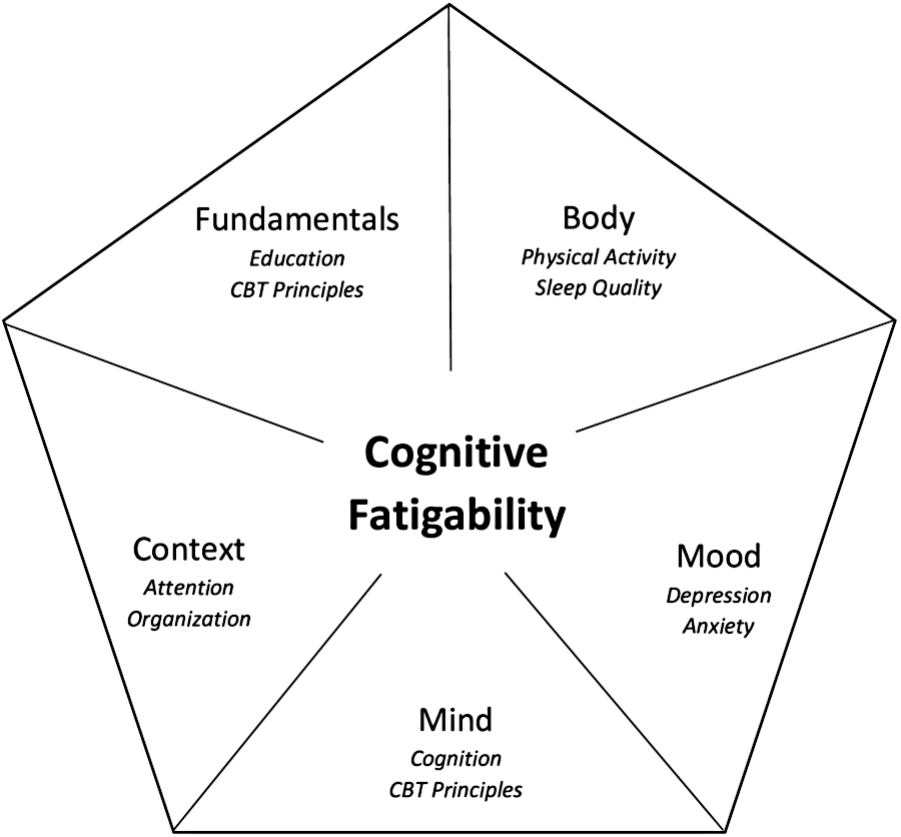
Intervention components.

The foundations for the currently proposed program stem from a previously successful 6-week program designed to target *subjective fatigue*. This new intervention will be expanded to a tailored 8-week videoconference-delivered program. This expansion will allow for the incorporation of additional elements more specific to *objectively measured CF* based on findings from our group's systematic review (54), our research team's work in this area, and theoretical principles. For example, a prediction model of CF highlighted the impact of both mood and sleep quality (38). As such, elements of Cognitive Behavioural Therapy (CBT) and CBT for Insomnia (CBT-I) will be incorporated (in consultation with a Clinical Psychologist) as these have previously been shown to be effective at addressing both mood and sleep dysfunction in MS. Furthermore, although there is no work to date that addresses the impact of exercise on objective CF specifically, a meta-analysis demonstrated that exercise training is an effective intervention for reducing subjectively measured fatigue in MS (48) and benefits on cognition have also been documented (63). Therefore, the tailored program will incorporate a physical activity component. Once all elements are incorporated, a facilitator manual will be prepared to ensure that the standardized intervention can be reliably administered across different facilitators and locations. To address the multidimensional nature of CF, the course material will focus on four different themes: *body* (contributions of sleep and physical activity), *mood* (impact of depression and anxiety on fatigue), *mind* (cognitive contributions), and *context* (pacing, communication). See Figure 2 for the intervention components. For the specific content covered in each of the 8 sessions, see Table 1.

**Table 1:** Intervention session content.

Session #	Session content
1	Understanding cognitive fatigability
2	Prioritization and goal-setting
3	Physical activity
4	Sleep, rest and pacing
5	Managing your mood
6	Strategies for improving cognition
7	Managing contextual factors
8	Review session

### Stage 2: Needs assessment

Although the initial iteration of the intervention plan will be established based on previous research findings and clinical experience of the investigators, it is important to consider the input of PwMS directly to ensure that we prioritize factors that they deem to be important contributors to their own CF. As such, we will conduct a needs assessment survey.

A need can be defined as a gap between the current condition and a desired condition (64). Democratic needs are typically determined by the preferences of the majority and can be identified by surveying the target population (64). A needs assessment is a tool that can consider those who have a stake in a situation, help to clarify the issues, set future goals, and provide data to guide future decision-making (64). This approach to addressing gaps in care has been used successfully in the past to ensure that the needs of PwMS are considered when designing a model of care in an MS Clinic (65, 66). In the current circumstance, a needs assessment can help to ensure that the behavioural intervention being developed is one that is relevant and will address the CF needs of those individuals for whom it is designed. While there are many types of needs assessment, the one utilized here will be a *strategic needs assessment* (64) that will allow us to identify the gaps in current treatment for those PwMS impacted by CF.

The survey will include a brief demographic questionnaire to identify the characteristics of our sample. The survey will also include previously validated measures assessing fatigue, as well as cognition, mood, sleep quality and physical activity so that we can determine to what extent the individuals surveyed are impacted by these factors (factors identified in the literature to contribute to CF). We will also include items to identify what contextual factors influence the fatigue of those surveyed (e.g., family responsibilities, etc.), as previous work has identified that addressing such contextual factors is an important component of a multi-dimensional fatigue intervention (60). See Table 2 for a list of the outcome measures that will be administered as part of the needs assessment. The survey will then address how *disruptive* these various challenges are to the individuals surveyed and they will be asked to identify whether they feel these areas are *sufficiently addressed* in both the currently available health care and community support systems. If an area is identified as disruptive and insufficiently addressed, then this will constitute an identified need and will help guide refinements made to the intervention.

**Table 2:** Questionnaires included in the needs assessment survey.

Area	Questionnaire
Fatigue	Modified Fatigue Impact Scale (77)
	Functional Assessment of Chronic Illness Therapy-Fatigue version (78)
	F-2-MS (78)
Cognition (self-report)	Perceived Deficits Questionnaire-5 (79)
Mood	Hospital Anxiety and Depression Scale (80)
Sleep Quality	Pittsburgh Sleep Quality Index (81)
Physical Activity	Godin Leisure Time Exercise Questionnaire (82)
Contextual Factors	Contextual Factors Affecting Functioning
Need Identification	Symptom Disruptiveness and Addressment Scale

We aim to survey a random sample of 100 PwMS. Following informed consent, respondents will be sent a secure link to a Qualtrics (67) survey that can be completed on a computer, tablet or smartphone at their convenience. Once data is compiled and analyzed, the information will inform potential modifications that may be needed to the proposed intervention plan. For example, if the survey were to reveal that contextual factors were less contributory to their CF than mood factors, then the intervention content addressing contextual factors could be diminished and the mood content could be further emphasized.

#### Data analysis for stage 2

Descriptive statistics will be compiled. We will determine what proportion of respondents rate the disruptiveness of each area of concern as high (i.e., they rate the degree of disruptiveness as a 4 or 5 on a Likert scale from 1 to 5). Similarly, we will determine what proportion of respondents rate the concern as being inadequately addressed (i.e., they rate the degree that the issues are addressed as 1 or 2 on a Likert scale from 1 to 5). This will then help inform how much emphasis should be given to each topic in the intervention. The more disruptive the issue and the less it is addressed with current resources, the greater emphasis it should have in the intervention. We will conduct a descriptive analysis to examine how those who indicate that CF is disruptive (i.e., 4 or 5 on the Likert scale) respond on questionnaires that reflect the themes covered in the intervention (i.e., mood, cognition, physical activity, context).

### Stage 3: Implementation of intervention

Stage 3 is the *implementation* phase and will begin with the training of an occupational therapist who will administer the manualized intervention. This individual will be trained by the Principal Investigator and/or Co-Principal Investigators. Any issues arising from this training will be identified and subsequent training will be modified accordingly. Given the additional mood component in this study, there is the potential for the identification of individuals with serious mental health concerns. As such, a psychologist (LW and/or a consulting Clinical Psychologist) will be available should the intervention of a mental health professional be required throughout the project. With respect to participants, we will recruit a sample of 20 individuals with MS who meet the inclusion criteria from the Ottawa Hospital MS Clinic. The sample size was selected so that we can ensure that we have 15 individuals in the final sample (allowing for 25% attrition). This number was chosen given that previous research has suggested a minimum sample size of 12 to 15 is sufficient to determine feasibility of pilot studies in MS (68, 69). The primary inclusion criteria is that participants exhibit evidence of objective CF on the PASAT as defined by established normative data using ≥1.5 standard deviations below the mean (36). Inclusion criteria also include: (a) English-speaking; (b) ages 18–65 years; (c) EDSS <6.0; (d) relapse and steroid free in the past 30 days; (e) purposeful exercise ≤2 days per week for 30 min; (f) asymptomatic (i.e., no signs or symptoms of acute or uncontrolled cardiovascular, metabolic, or renal disease) based on the Get Active Questionnaire (70), (g) sufficient visual function to complete cognitive tasks (e.g., no scotomas) and (h) access to an internet-enabled device to participate in the intervention. Exclusion criteria are: (a) other neurological, medical or psychiatric condition that might impede cognition (e.g., traumatic brain injury, learning disability) excluding depression and anxiety; (b) current dementia; (c) substance use or dependence disorder; (d) hearing impairment that would interfere with the ability to effectively take part in the videoconference sessions.

The research assistant will be available to perform participant screening on the inclusion criteria, the Get Active Questionnaire and the PASAT during all MS Clinics until the sample size is reached. For those meeting eligibility requirements, a baseline assessment session (lasting approximately 120 min) will take place at the Ottawa Hospital for each participant with outcome measures administered by a research assistant (see Table 3). These outcomes measures were chosen given the multidimensional nature of fatigue in MS, and given that these variables are, or have the potential to be, related to CF. The pre-intervention assessment will take place at least 1 week before the beginning of the intervention to ensure that participants can wear an accelerometer (to monitor physical activity) for at least 1 week to establish baseline physical activity. The 8-week, group-based intervention will involve weekly 70-min videoconference calls facilitated by the licensed occupational therapist (with sessions reviewed by the psychologist). Given the sample size, we anticipate three separate groups (i.e., no more than 7 per group). These three groups will be provided at different times of day to allow scheduling flexibility for participants. The facilitator will promote discussion within group members by calling on individual participants during the calls to allow for interaction, social learning and peer support. Homework will be a component of the program and will be reviewed at each subsequent session. Abbreviated make-up sessions will be offered on an as-needed basis for participants who need to miss a class. Completers will be defined as individuals who attend at least 6/8 sessions (i.e., 75%). After each session, the facilitator will complete SOAP notes (subjective, objective, assessment, plan) to document their impressions (71). Within 2 weeks of the last videoconference session, participants will return to the Ottawa Hospital for a follow-up assessment session (∼120 min) where outcome measures (using alternate forms where possible) will again be administered by a research assistant.

**Table 3:** Outcome measures for the behavioural intervention.

Outcome	Measures
Cognitive fatigability	Paced Auditory Serial Addition Test (83), Psychomotor Vigilance Task (84), F-2-MS (78)
Subjective fatigue	Modified Fatigue Impact Scale (77), Fatigue Scale for Motor and Cognitive Functions (16), Fatigue Symptoms and Impacts Questionnaire-Relapsing MS (17)
Cognition	Brief International Cognitive Assessment for MS (Symbol Digit Modalities Test (85), learning trials of the California Verbal Learning Test-II (86) & Brief Visuospatial Memory Test-Revised) (87), Phonemic and semantic fluency (88)
Mood	Hospital Anxiety and Depression Scale (80), Patient Health Questionnaire-9 (89) Generalized Anxiety Disorder-7 (90) Depression Anxiety and Stress Scale (91)
Health-related quality of life	Multiple Sclerosis Quality of Life (MSQOL)-54 (92)
Sleep quality	Pittsburgh Sleep Quality Index (81)
Physical	Free-living activity using accelerometer (ActiGraph GT3X-BT; processed using ActiLife software) (www.actigraphcorp.com)
Self-efficacy	Likert scale (93) regarding confidence in ability to manage MS fatigue
Structured Interview	Structured interview with participants regarding knowledge and attitudes toward the intervention (quantitative based on Likert ratings) with items reflecting the seven component constructs of the Theoretical Framework of Acceptability (94)
Feasibility	Eligible participants excluded or not agreeing to participate; completion rate of assessments; attendance at intervention sessions; adherence to homework; attrition; facilitator SOAP notes

### Stage 4: Analysis and dissemination

Stage 4 involves *analysis and dissemination of results*. Data will first be compiled and analyzed using IBM SPSS Statistics (version 28). Descriptive statistics will be compiled to characterize the sample. Comparisons between baseline and follow-up on the outcome measures for all completers will be conducted using *t*-tests. The primary outcome measure is feasibility and will be evaluated according to the following: eligible participants excluded or not agreeing to participate, completion rate of assessments, attendance at intervention sessions, adherence to homework, attrition, and facilitator SOAP notes. The secondary outcome measure is CF performance on the PASAT. CF scores will be derived according to previously documented procedures (36). The intervention will be considered effective if at least a small effect size (i.e., improvement in CF) is observed between baseline and follow-up. Data from the structured interview will be analyzed quantitatively. Facilitator SOAP notes will be subjected to thematic analysis. Knowledge translation will involve traditional dissemination routes (i.e., journals, conferences) with the additional plan to disseminate this research among health care professionals by providing in-service presentations and hospital rounds. We will also seek to disseminate the findings to the lay public through community presentations, and educational materials. It is our intention to include trainees at all levels in this research to educate new health care professionals on the benefits of behavioural interventions on patient outcomes. We will also engage a Patient Advisory Committee to seek input on other possible avenues of communicating the results from this study to individuals in the MS community. We will follow CONSORT reporting guidelines for pilot feasibility trials (72).

### Stage 5: Refinement

Stage 5 is the *refinement* phase that involves modification of the behavioural intervention based on the outcome measures, the feedback from the participants, as well as input from the Patient Advisory Committee. The goal of this stage is to prepare for and plan a future RCT to definitively evaluate the efficacy of the intervention based on the CF findings.

Table 4 provides a graphical timeline of the proposed study.

**Table 4:** Study timeline.

Study period							
	Stage 1	Stage 2	Stage 3			Stage 4	Stage 5
Study phases	Development	Needs Assessment	Pre-intervention	Intervention	Post-intervention	Analysis & Dissemination	Refinement
Timeline	6 months	12 months	12 months	3 months	3 months
Development of intervention	X						
Preparation of facilitator manual	X						
Recruitment for Stage 2		X					
Enrolment for needs assessment survey		X					
Informed consent for survey		X					
Survey administration		X					
Survey data analysis		X					
Intervention modification		X					
Facilitator manual modification		X					
Training of occupational therapist			X				
Recruitment for Stage 3			X				
Eligibility screening			X				
Enrolment for intervention			X				
Informed consent for intervention			X				
Outcome measures							
PASAT			X		X		
PVT			X		X		
MFIS			X		X		
FSMC			X		X		
FSIQ-RMS			X		X		
BICAMS			X		X		
Phonemic fluency			X		X		
Semantic fluency			X		X		
HADS			X		X		
PHQ-9			X		X		
GAD-7			X		X		
DASS			X		X		
MSQOL-54			X		X		
PSQI			X		X		
Actigraph			X		X		
Structured interview			X		X		
Biomarkers			X		X		
Intervention sessions 1 through 8				X			
Facilitator SOAP notes				X			
Feasibility outcomes					X		
Completion rate of assessments					X		
Attendance at intervention sessions					X		
Homework adherence					X		
Attrition					X		
Facilitator SOAP notes completion					X		
Data analysis						X	
Knowledge translation						X	
Refinement							X
Future RCT planning							X

ActiGraph, ActiGraph Free-living activity accelerometer; BICAMS, Brief International Cognitive Assessment for Multiple Sclerosis; DASS, Depression Anxiety and Stress Scale; FSIQ-RMS, Fatigue Symptoms and Impacts Questionnaire-Relapsing MS; FSMC, Fatigue Scale for Motor and Cognitive Functions; HADS, Hospital Anxiety and Depression Scale; MFIS, Modified Fatigue Impact Scale; MSQOL-54, Multiple Sclerosis Quality of Life; PASAT, Paced Auditory Serial Addition Test; PHQ-9, Patient Health Questionnaire-9; PSQI, Pittsburgh Sleep Quality Index; PVT, Psychomotor Vigilance Task.

## Discussion

There is a recognized need for behavioural interventions targeting the improvement of CF given the negative implications for quality of life (24, 25) in approximately half of PwMS (32). Few interventions exist to date, with pharmacological approaches being unsuccessful (57), and procedural interventions being inaccessible to most PwMS (56). Our own work has demonstrated the promise of behavioural interventions (60) and that there are multiple contributing factors to CF that must be considered when designing such an intervention (38). The proposed pilot study addresses questions of *feasibility*, as well as a specific design feature where we test the *efficacy* of the intervention on a smaller scale in preparation for a future RCT (62).

This study has several strengths. Collectively, our interdisciplinary team has the qualifications and resources to carry out this project, and team leads have extensive clinical and research experience in MS. Our team has prior skills in conducting needs assessments and in executing rehabilitative interventions targeting both cognition and fatigue. Collectively, our team has expertise in neuropsychology (LW), occupational therapy (MF), neurology (SM), exercise physiology (LP), and experimental psychology (JB). Our team has many years of both clinical and research experience in the field of MS. The Needs Assessment and Intervention will be designed based on both the scientific literature and the knowledge we have gained in working directly with those affected by MS. The intervention will be informed by theoretical principles and known best practice standards. LW, JB and SM have established validated measures for assessing CF in MS (23, 26). LW and JB have developed predictive models of CF (38), have established functional neuroimaging biomarkers of CF (29), and were the first to establish normative data for CF in MS so that findings can be put into clinical practice (36). SM has investigated pharmacological treatments for CF (57). MF is known internationally for her interventional studies designed to reduce fatigue in MS (60). LP, a recognized expert in exercise interventions in MS (73), will provide specific input into the exercise aspect of the intervention and associated outcomes. In addition to JB providing his CF expertise (38), he will also lend statistical support to the project. The study will take place in Ottawa where the MS Clinic serves over 3,000 active patients with MS. Physical resources and organizational support will be provided by the Ottawa Hospital Research Institute and the University of Ottawa Brain and Mind Research Institute.

Our group has conducted numerous studies before and thus do not anticipate any difficulties recruiting participants for either the Needs Assessment or Intervention components. We anticipate that recruiting individuals interested in the CF intervention will not be challenging given that this is an issue that affects approximately half of all individuals with MS (74). However, the process of screening interested individuals is likely to take the most time at this stage given that not all those expressing interest will meet eligibility criteria, particularly as it relates to objectively measured CF. This will require that a research assistant be present at MS Clinics so that screening can occur as soon as potential participants are identified. A graduate student (TI) will be assigned to this task given that the work will form part of the student's dissertation. As such, this is not expected to lead to any significant barriers.

An additional advantage of this study is that we are able to use technology to conduct both the needs assessment (i.e., internet-delivered survey) and intervention (i.e., internet accessible videoconference software) components. This limits face-to-face contact; a feature of this research that has become more necessary in the context of a global pandemic. While there is the possibility that a resurgence of COVID-19 could limit our ability to bring in participants for the pre- and post-intervention assessment sessions, this portion of the study is scheduled for 1 year in the future. As full lockdowns become less common, and hospitals are now better equipped to provide appropriate PPE and accommodations (e.g., use of a plexiglass screens, etc.), we do not anticipate that increased COVID-19 in the community will cause any significant study-related delays.

A further strength of this project is the inclusion of a needs assessment survey to ensure that the needs of PwMS are considered. Such a survey allows PwMS to identify the particular factors that they feel are important regarding their own CF and informs the development and refinement of the intervention. By taking into account the concerns identified by PwMS, we can be sure that the intervention will be tailored to meet their needs.

A potential limitation of any computer-based delivery model is that they have the potential to limit accessibility for those who are not confident in their technological skills or do not have access to the required hardware. However, the platforms we have chosen can both be accessed by smartphones. Although less ideal in terms of format, most individuals have access to smartphones and thus accessibility is not expected to be a barrier for participants.

The multidimensional nature of the intervention has both strengths and perceived limitations. By addressing multiple underlying components contributing to CF, the intervention can be tailored to meet the needs of each individual. This strength allows the intervention to address multiple factors that potentially contribute to CF in any given individual. While some might suggest that a multidimensional intervention does not easily allow for the identification of the most efficacious component, it is important to acknowledge that CF itself is a multidimensional symptom and what proves to be most efficacious for one individual may differ for another. While one person may find that increasing their physical activity improves their CF, another may find that addressing their mood concerns is of more benefit. So too might the factors contributing to CF in one individual change over time, with contextual factors being of more importance in 1 week and mood factors being more contributory in the next. The multidimensional nature of the intervention allows for the required flexibility to address these changing needs both between and within individuals.

We anticipate several outcomes from this project. First, we expect that the modifications identified by the needs assessment will be easily implementable into the planned intervention and associated facilitator manual. During the initial development in Stage 1 the intervention will be designed with these things already in mind. It is expected that each of the areas included in the needs assessment will be indicated as disruptive and currently unaddressed by at least some individuals. We anticipate that respondents will identify several areas of unmet need, particularly as they relate to treatments for fatigue, mood, and cognitive challenges. Our own group has identified a significant gap between the research progress in these areas and the services available to address these issues in standard clinical practice (75). While exercise interventions may be more readily available in the community at large, there continue to be environmental barriers to accessibility (76–78). The current intervention will be designed to address each of these components (i.e., body, mood, mind, context), highlighting once again, the flexibility of such an intervention. Second, we anticipate that at least 75% of the individuals enrolled in the intervention will complete 6/8 sessions (i.e., be successful completers). While attrition is anticipated in any intervention study, past studies have demonstrated that having at least 12 completers is sufficient to yield the necessary outcomes in a pilot study (68). Third, we anticipate that those completing the intervention will demonstrate an improvement in CF as measured by a positive and statistically significant change documented from baseline assessments. Fourth, taken together, the needs assessment and intervention are expected to inform the development of a future definitive RCT. If such an RCT were to be successful, then further RCTs to compare the efficacy of the behavioural intervention to procedural interventions (i.e., tDCS) and pharmacological interventions could be pursued.

The ultimate goal of this multi-staged project is to ensure that those with MS have access to effective interventions in real-world settings to improve the quality of their lives and enhance their ability to participate in cognitively demanding activities that they enjoy.

## Data Availability

The original contributions presented in the study are included in the article/Supplementary Material, further inquiries can be directed to the corresponding author/s.
